# Self-protective behaviors of Thai village health volunteers in community engagement during a COVID-19 outbreak: interpretation using the health belief model

**DOI:** 10.1186/s12875-024-02346-z

**Published:** 2024-03-28

**Authors:** Paleeratana Wongrith, Phuwasin Buakate, Lateefah Doylee, Naseeyah Phonla, Omid Dadras, Geoff Frampton

**Affiliations:** 1 School of Public Health, Walailak University, 222 Thaiburi, Tha Sala, Nakhon Si Thammarat, 80161 Thailand; 2 Department of Global Public Health and Primary Care, University of Bergen, Bergen, Norway; 3 The Excellence Center Dengue and Community Public Health (EC for DACH), Walailak University, Tha Sala, Nakhon Si Thammarat, Thailand; 4 Southampton Health Technology Assessments Centre (SHTAC), Faculty of Medicine, University of Southampton, Southampton, UK

**Keywords:** Community engagement, Health belief model, Self-efficacy, Self-protective behavior, Village health volunteers

## Abstract

**Background:**

Village health volunteers (VHVs) engaging in community-based COVID-19 prevention and control measures played a key role in mitigating effects of the COVID-19 pandemic in Thailand. We conducted a cross-sectional questionnaire survey study to investigate factors affecting VHVs’ COVID-19 self-protective behaviors and social distancing in Songkhla Province during the first COVID-19 outbreak. Such information may help to understand how to support VHVs in future pandemics.

**Methods:**

A total of 152 VHVs from 13 sub-districts participated in the study, completing a 54-item questionnaire based on the Health Belief Model (HBM). The questionnaire included items assessing susceptibility, severity, benefits, barriers, self-efficacy, social distancing, and self-protective behavior. Stepwise multiple regression analysis determined which aspects of the HBM could explain VHVs’ self-protective behavior.

**Results:**

The VHV population sampled broadly reflected the main demographic characteristics of the local population, although VHVs were predominantly female. Self-protective behavior was significantly associated with VHVs’ role (higher perceived compliance for village leaders than non-leaders) but not with other demographic characteristics. Most VHVs reported high levels of self-efficacy (80.5%), adherence to social distancing measures (70.9%), and engagement in self-protective behavior (72.8%) against COVID-19. However, compliance with hand hygiene appeared to be suboptimal, suggesting room for improvement. Self-efficacy and perceived social distancing showed strong and moderate correlations with self-protective behavior against COVID-19 (*r* = 0.917, β = 0.819; and *r* = 0.561, β = 0.173 respectively; *p* < 0.001). The final HBM-based regression model accounted for 87.2% of the variance in VHVs’ self-protective behavior.

**Conclusions:**

This study highlights the importance of VHVs’ self-efficacy for achieving self-protective behavior during a COVID-19 outbreak, and suggests that self-efficacy may help to overcome barriers that might otherwise hinder behaviors to mitigate against COVID-19. Policies that support self-efficacy should be implemented in any future pandemic, and steps to support VHVs with hand hygiene compliance and empower non-leaders to increase their self-protective behavior may also be helpful. Whilst the HBM provided a useful framework for interpretation, the final model was driven mainly by self-efficacy.

**Supplementary Information:**

The online version contains supplementary material available at 10.1186/s12875-024-02346-z.

## Background

The WHO declared the COVID-19 epidemic a Public Health Emergency of International Concern [1, 2]. Thailand has been significantly impacted, with over 4.3 million confirmed cases and 28,976 deaths during the first-wave period from January 2020 to July 2020 [2, 3]. The success of COVID-19 public health measures depends heavily on public compliance [4]. The community engagement structures of community organizations and local leaders show how communities play important and active roles in infection prevention and control [3, 5]. The Thai government established the Center for COVID-19 Situation Administration (CCSA) as a single command center on March 12, 2020, to ensure clear communication and a consistent understanding of the situation [3, 6]. To minimize the impact of COVID-19, various strategies including testing, isolation, contact tracing, quarantine, and community involvement, have been implemented to reduce mortality, morbidity, and economic losses [7].

Village Health Volunteers (VHVs) have been a key part of primary health care in Thailand during the past four decades [8, 9]. VHVs played a crucial role in preventing the spread of the SARS-CoV-2 virus by screening at-risk individuals, monitoring home visits, conducting awareness campaigns, and assisting in contact tracing, testing, and quarantine measures [10, 11]. However, they faced several challenges, such as lack of personal protective equipment (PPE), misinformation and stigma, language barriers, and lack of recognition and support [5, 12]. To reduce exposure to the SARS-Cov-2 virus, Centers of Disease Control (CDC) guidelines recommended a 6-foot (1.82 m) social distance, working from home, avoiding travel, and avoiding large gatherings and crowding in public places [13]. VHVs and the public were trained to practice social distancing and avoid public places and contact with high-risk individuals by the health providers [11, 14]. VHVs in Thailand were required to follow CDC recommendations for standard precautions, such as hand hygiene, PPE, respiratory hygiene, and cough etiquette [4, 6]. Additionally, depending on their roles, VHVs participating in COVID-19 screening had to adhere to specific standard precautions [10, 15].

The Health Belief Model (HBM), developed by Becker in 1974, consists of perceived susceptibility, severity, benefits, and barriers. In 1988, self-efficacy was incorporated into the HBM to further understand individual perceptions [16]. This model has been extensively utilized in previous studies to explain individual beliefs regarding health. Perceived susceptibility and perceived severity are related to negative outcomes and an individual’s perception of risk, while perceived benefits and perceived barriers relate to positive and negative consequences respectively of adopting preventive behavior [17, 18]. Self-efficacy is a person’s belief in their ability to carry out a specific action [19, 20].

The HBM has been applied to predict the use of PPE and risk determinants during previous SARS outbreaks [21, 22]. It has also been used to examine other preventive behaviors related to COVID-19, such as wearing masks, handwashing, physical distancing, and following standard precautions [19, 23].

Several factors can influence people’s acceptance of COVID-19 guidelines and participation in protective behaviors, including age, gender, marital status, education, and HBM perception of COVID-19 [23, 24]. For example, studies have shown that female sex, older age, and higher education status are positively associated with compliance to COVID-19 protective behaviors and safety precautions, such as social distancing and hand washing [25]. The effectiveness of VHVs in achieving COVID-19 protective behaviors could also depend on effective communication and delegation of responsibilities between leaders and VHVs [24, 26]. In a study by Tejativaddhana et al. the HBM components including perceived susceptibility, severity, barriers, benefits, and self-efficacy were suggested as crucial components of COVID-19 self-protective behavior in Thailand [4]. In addition, Boonchailert et al. [8] and Chanarnupap et al. [14] studied the role of guidelines for competency development among VHVs dealing with emerging infectious diseases in Thailand. Several studies have also explored the role of guidelines for VHVs in dealing with emerging infectious diseases and applied the HBM in community engagement efforts to explain the public’s adopted prevention practices during the COVID-19 outbreak [22]. Designing an educational intervention based on the HBM could effectively guide correct beliefs and promote adherence to COVID-19 preventive behaviors [17, 18, 27]. Nevertheless, limited research has focused on the self-protective behaviors of public health volunteers during the first wave of the COVID-19 outbreak in Southern Thailand. In this study we investigated how Village Health Volunteers (VHVs) in Southern Thailand perceive COVID-19 protective measures, using the Health Belief Model (HBM) framework within the Health District system. There is limited research on VHVs’ community engagement during the early stages of the pandemic, despite their crucial role in strengthening public health, particularly amid challenges like resource constraints, misinformation, and inadequate facilities caused by COVID-19. By addressing this gap, our study aims to offer insights that can improve VHVs’ responses in future outbreaks, leading to more effective public health strategies.

## Methods

### Study design

A cross-sectional study was conducted between August 1, 2020, and September 30, 2020, during the first wave of the COVID-19 pandemic in Chana District, Songkhla Province, Southern Thailand. The study utilized a pre-piloted questionnaire to assess the self-protective behaviors of Thai Village Health Volunteers (VHVs) against COVID-19 and evaluate their concordance with the Health Belief Model (HBM) components.

### Population and sample

A total of 1,463 registered VHVs from 14 subdistricts in Chana District were initially considered for recruitment (for details of the recruitment sites see Appendix A). One subdistrict with 48 VHVs was excluded from the main survey as it was selected for the pilot study. The pilot study included 30 randomly selected VHVs from the corresponding subdistrict. The remaining 1,415 VHVs from 13 subdistricts were eligible for potential recruitment based on the inclusion and exclusion criteria.

Inclusion criteria: To participate in this study, VHVs must meet the following criteria:

1. According to the requirements of the Ministry of Public Health, VHVs involved in COVID-19 outbreaks must be 20–60 years old.
2. They must have received COVID-19 screening training from the Health District System (DHS).
3. Proficiency in online communication using smartphones and familiarity with the use of the VHVs group Line app.
4. VHVs should be willing to participate in research activities and provide informed consent.

Exclusion criteria: VHVs were excluded from the study if they did not participate in screening during the specified period of the COVID-19 outbreak, did not complete the questionnaire, or felt uncomfortable while answering the questionnaire.

### Sample size and recruitment process

Using the G*Power program, a sample size of 138 was calculated based on a moderate correlation (Cohen’s estimate, *r* = 0.30) [28], an α level of 95%, and a 2-sided type I error rate of 0.05. To account for potential dropout, 152 participants were recruited. VHVs who met the inclusion criteria were identified and listed in each subdistrict. A total of 152 participants were randomly selected proportionally to the subdistrict size, with 10–20 VHVs per subdistrict.

### Research instruments

Data were collected using a self-reported questionnaire specifically developed for Thai VHVs. The questionnaire comprised 54 questions covering sociodemographic characteristics, HBM variables, and self-protective behaviors related to COVID-19 community engagement. The questionnaire was written and communicated in the Thai language (for an English translation of the questions see Appendix B). The questionnaire addressed the following topics:

- Demographic information (VHVs’ baseline characteristics): Seven questions (Q1-Q7).
- Perceived susceptibility to COVID-19: Six questions (Q8-Q13).
- Perceived severity of COVID-19: Five questions (Q14-Q18).
- Perceived benefits of COVID-19 self-protection: Five questions (Q19-Q23).
- Perceived barriers to protection from COVID-19: Seven questions (Q24-Q30).
- Perceived self-efficacy (a person’s confidence and ability to protect themselves from COVID-19): Seven questions (Q31-Q37).
- Perceived social distancing (this was a mandatory part of the Government’s COVID-19 protection strategy): Six questions (Q38-Q43).
- Compliance with standard precautions other than social distancing against COVID-19 infection: Six questions (Q44-Q49).
- Compliance with the Thailand Stop-COVID-19 policy: Five questions (Q50-Q54).

Questions 1–7 each have either two or three alternative nominal responses. Questions 8–43 score participants’ compliance on a 3-point Likert scale with responses ranging from 1 (least agreement) to 3 (strongest agreement). Questions 44–54 assess VHV’s self-protective behavior in terms of compliance with standard practices on a 3-point Likert scale ranging from “Sometimes practicing” which refers to practicing no more than two days per week or not practicing at all (1 point); “Practicing often” which indicates practicing 3–5 days a week (2 points); and “Always practicing” which indicates practicing 6–7 days per week (3 points). The questionnaire’s relevance, word choice, content, and comprehensiveness were reviewed by a team of three experts (Appendix C). Items with an Index of Objective Congruence (IOC) of 0.75 or higher were included in the questionnaire. The questionnaire’s internal consistency was assessed through a pilot study with 30 VHVs, resulting in a Cronbach’s α of 0.83, indicating good internal consistency.

### Data collection

Two trained assistant researchers collected data from all recruited VHVs in Chana District. Data collection took place at subdistrict health centers from August 1 to September 30, 2020. Each interview lasted an average of 30 min duration.

### Ethical considerations

The protocol of the present study was reviewed and approved by the Ethics Committee of Walailak University (certificate number WUEC-20-204-01) on 30 July 2020. Participants were informed that their participation in the study was entirely voluntary and confidential, and they had the right to withdraw from the study at any time. Each participant signed a consent form after receiving full information about the purpose and methods of the study.

### Data analysis

We conducted three analyses to achieve the following objectives: (1) determining the association between VHV’s COVID-19 self-protective behaviors and participant demographic characteristics, (2) exploring the relationships among the primary elements of the HBM (susceptibility, severity, benefits, barriers, self-efficacy) and their connection to social distancing and self-protective behavior, and (3) investigating which elements of the HBM can explain VHV’s COVID-19 self-protective behavior.

### Analysis 1: demographic variables and self-protective behavior(SPB)

We examined the relationships between the total self-protective behavior score and demographic variables using scatter plots for continuous variables (age, income) and subdistrict (13 classes). For categorical variables (sex, religion, education, VHV role, and occupation), we utilized contingency tables and conducted a Chi-square test. The scatter plots were scaled to capture the possible range of the total SBP score across the 11 self-protective behavior questions (questions 44 to 54), with terciles used to differentiate total scores corresponding to “sometimes practice”, “often practice”, and “always practice”. Due to the limited number of VHVs falling into the “sometimes practice” category, contingency tables were constructed to compare “full SBP compliance” (the “always practice” response category) against “partial compliance” (the combined response categories “sometimes practice” and “often practice”) to explore relationships with categorical demographic variables.

### Analysis 2: relationships between HBM elements, social distancing, and self-protective behavior 

We examined the frequencies of VHV responses per question (on the original 3-point Likert scale) using histograms to visualize response patterns within and between the susceptibility, severity, benefits, barriers, self-efficacy, social distancing, and self-protective behavior categories. Additionally, we conducted a Pearson correlation analysis (SPSS version 25.0) to investigate associations among these variables. A correlation coefficient greater than 0.8 was considered strong, and statistical significance was set at 0.05.

### Analysis 3: analysis of HBM factors explaining self-protective behavior 

Stepwise multiple regression (backward selection) (SPSS version 25.0) was performed with self-protective behavior as the dependent variable and the five HBM elements (susceptibility, severity, benefits, barriers, self-efficacy) and social distancing as independent variables. This method starts with a model that includes all variables and then removes the least significant variable at each step until no more variables meet the criteria for removal. The total scores for each variable followed a normal distribution and met the normality assumption (Appendix D) [29–31]. The normality of the regression residuals was confirmed using normal probability plots. The most significant independent variables that yielded the best fit were selected to build the final regression model. Model fit and multicollinearity were assessed using relevant statistics and post-estimation tests. Variables with a variance inflation factor > 5 were excluded. R^2^ values were reported to explain the proportion of variation in the dependent variable explained by the final model, with R^2^ values above 0.8 considered high, 0.5–0.79 considered moderate, and below 0.5 considered low.

## Results

All 152 VHVs completed the questionnaire with no missing data. The mean age of the participants was 49.2 (range 20–60) years, most (84.2%) of the participants were female, 64.5% were Muslim, 66.4% worked as farmers, and half (50%) had completed high school. In terms of monthly income, nearly half of the participants (48%) earned between 5,001 and 10,000 Baht per month. Most participants (77.6%) had the role of a village leader, and 87.5% had a high perception of COVID-19 self-protective behavior.

### Relationship between self-protective behavior and demographic variables

The total self-protective behavior score of VHVs was significantly associated with the role of VHVs (*p* < 0.05) (higher for village leaders than non-leaders) but not with any of the other demographic variables assessed (Table 1; further details in Appendix E). Overall, the majority of VHVs (87.5%) had total SPB scores in the upper centile (always practiced), 11.8% had scores in the middle centile (often practiced) and just one VHV had a score in the lower centile (sometimes practiced). All 19 VHVs who had total SBP scores below the upper centile were aged above 40 years. However, only a small proportion of the VHVs (11.8%) were aged 40 years or less, so any association between age and SPB is not conclusive. Four of the subdistricts (numbered 11–14), Baan Na, Pa Ching, Sakom, and Saphan Maikan, had relatively narrow ranges of total VHV self-protective behavior scores which lie within the upper centile of total scores (always practiced), indicating that all VHVs in those subdistricts had high compliance with self-protective behaviors. These four subdistricts are separated geographically (Appendix A) so appear unlikely to have been influenced by a common geographical factor that would not also have affected the other subdistricts. The high compliance in these four subdistricts is not clearly explained by an effect of age since these subdistricts did not contain many VHVs aged under 40 years.

**Table 1:** Association between self-protective behavior and sociodemographic characteristics of VHVs

VHVs Demographic		Total n(%)	Partial SPB compliance^a^	Full SPB compliance^b^	*p*-value
Sex	Male	24 (15.8)	5	19	0.179
	Female	128 (84.2)	14	114	
Religion	Buddhist	54 (35.5)	3	51	0.055
	Muslim	98 (64.5)	16	82	
Education	Primary school only	70 (46.1)	10	60	0.539
	Beyond primary school	82 (53.9)	9	73	
Role	Village leader	118 (77.6)	11	107	0.027
	Non-leader	34 (22.4)	8	26	
Occupation	Self-employed	28 (18.4)	7	21	0.085
	Farmer	101 (66.4)	2	21	
	Other employment	23 (15.1)	10	91	

Note. ^a^Partial SPB (self-protective behavior) compliance: sometimes/often practice, *n* = 19, ^b^Full SPB compliance: always practice, *n* = 133

### Health belief model and self-protective behavior among VHVs

**Fig. 1:**
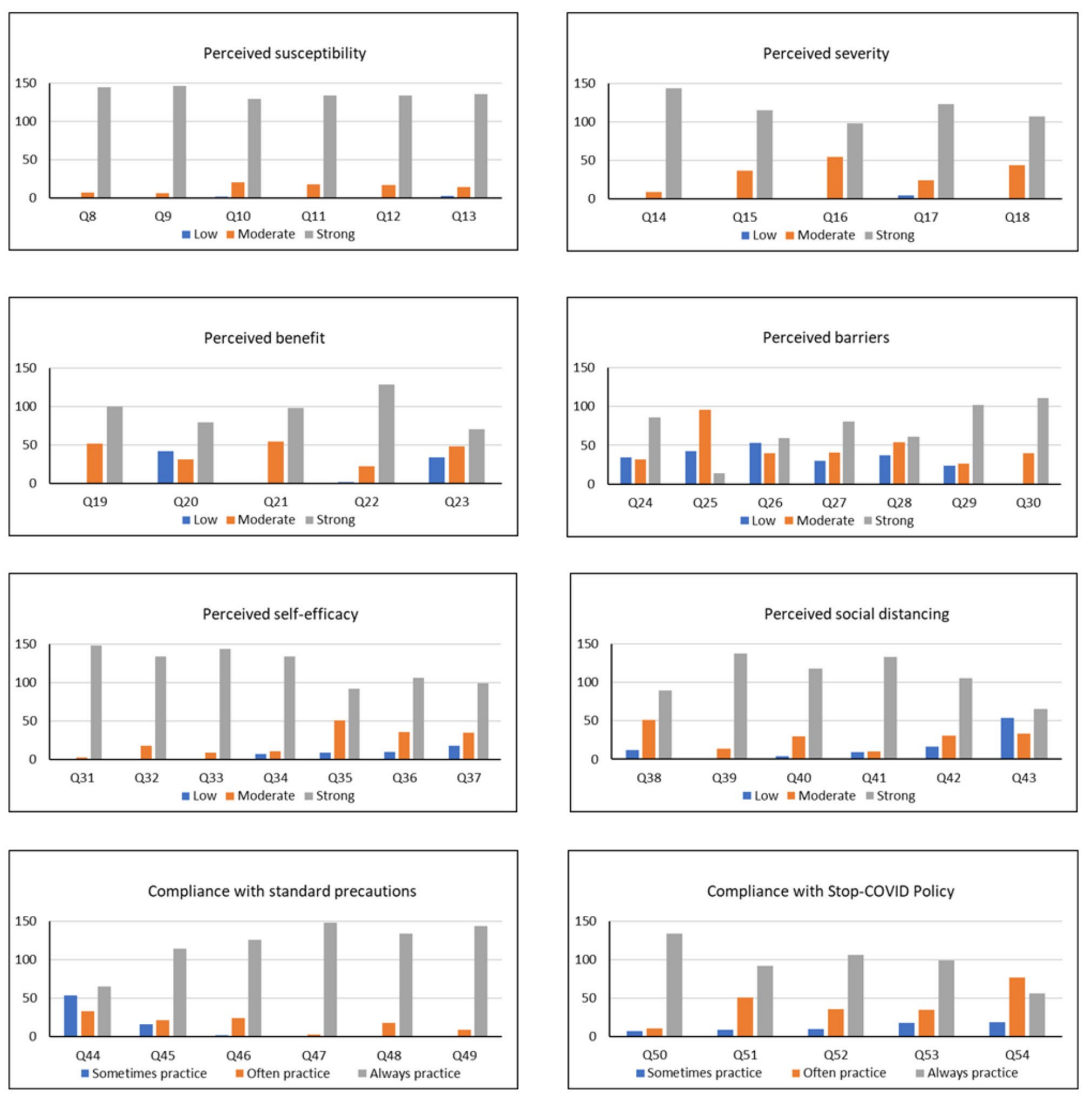
Frequency of VHV responses per question (*N* = 152) (for questions see Appendix B)

The frequencies of VHVs’ responses for each question are summarized in Fig. 1. Most VHVs had a high agreement on perceived susceptibility (90.1%) and perceived severity (77.9%). The majority of VHVs also indicated high agreement on the benefits of COVID-19 self-protection (62.5%). However, less than half of the VHVs (48.3%) had high agreement on barriers to protection from COVID-19 such as the inconvenience of masks (Q24), stress of quarantine (Q26), inconvenience of staying at home (Q27) and holding meetings online (Q29), with varying degrees of perception on the inconvenience of hand washing as a barrier (Q25, Q30).

As shown in Fig. 1, despite the perceived barriers, VHVs’ self-efficacy to practice self-protective behaviors was high, with 80.5% of VHVs expressing strong agreement with the self-efficacy questions and only 4.2% expressing low agreement. Most VHVs (70.9%) also agreed strongly with social distancing measures for COVID-19 self-protection, although this was not universal, with 10.5% expressing poor agreement, which seems to be consistent with some social distancing concerns reflected in Q20 about social distancing being perceived as a barrier by some VHVs. Among the social distancing questions, there was less agreement among VHVs for the need to take time off work after contacting a person with COVID-19 (i.e., post-event rather than prophylactic social distancing).

Compliance with standard precautions was rated as high, with 80.0% of VHVs across all the compliance questions responding that they “always practice” standard precautions and only 8.0% reporting that they “sometimes practice”. The “sometimes practice” responses appear to be mostly related to Q44 and Q45 (Fig. 1). Q44 specifically concerns handwashing after touching a person who is at risk of being infected with COVID-19 (as opposed to touching a person who has confirmed COVID-19), whilst Q45 requires compliance with all seven steps of the hand-hygiene routine. Questions about compliance with the Thailand Stop-COVID policy were rated high (“always practice”) by 64% of VHVs, with just over one-quarter of VHVs (27.6%) reporting they “often practice” the Stop-COVID policy elements and only 8.3% reporting that they “sometimes practice”. Taking the standard precautions and Stop-COVID policy together (i.e. based on 11 questions (Q44 to Q54), VHVs reported high overall compliance with self-protective behavior: 72.8% reported that they “always practice”, 19.1% that they “often practice” and 8.1% that they “sometimes practice”.

### Relationship between health belief model components and VHVs’ self-protective behavior

**Fig. 2:**
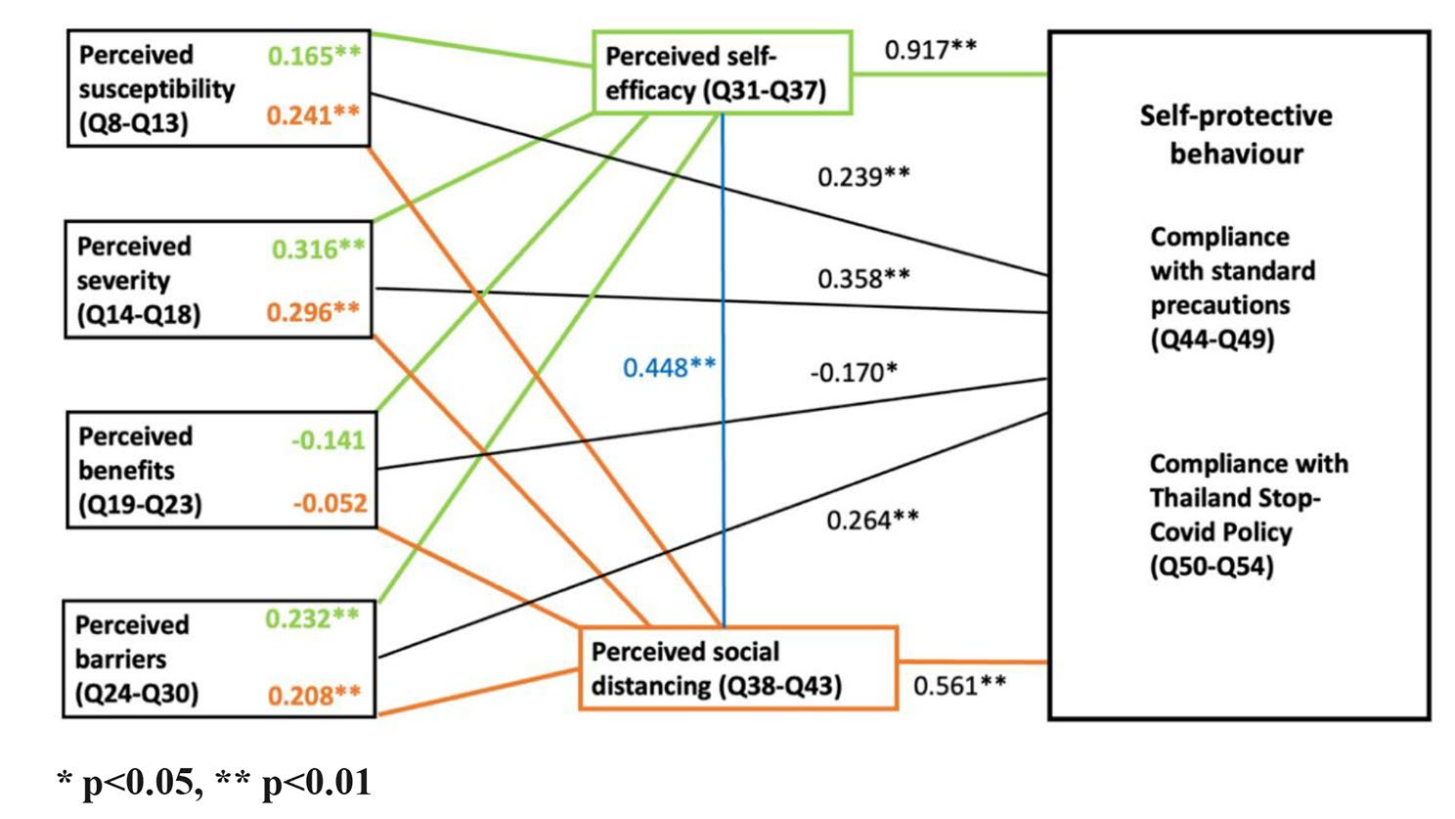
Correlation between Health Belief Model Components and VHVs’ self-protective behavior

The results of the correlation analysis are summarized in Fig. 2 (full correlation matrix in Appendix F).

Overall, correlations among the elements of the HBM and social distancing were generally weak except for perceived self-efficacy which was strongly correlated with self-protective behavior (*r* = 0.917, *p* < 0.001). Perceived social distancing showed a moderate correlation with self-protective behavior (*r* = 0.561, *p* < 0.001) and with perceived self-efficacy (*r* = 0.448, *p* < 0.001).

The stepwise regression analysis results indicate that perceived self-efficacy was strongly and positively associated with COVID-19 self-protective behavior (β = 0.819, *p* < 0.001), while social distancing was also significantly but less strongly associated with self-protective behavior (β = 0.173, *p* < 0.001). In the final model, self-efficacy, social distancing, perceived susceptibility, and perceived benefit explained 87.2% of the variation in the COVID-19 self-protective behavior (Table 2).

**Table 2:** The association of the Health Belief Model associated and COVID-19 self-protective behavior among participants *N* = 152

HBM components	Standardized Coefficients		95.0% Confidence Interval for Beta	
	Beta	Std. Error	Lower Bound	Upper Bound
Self-efficacy	0.819	0.047	1.076	1.262
Social distancing	0.173	0.050	0.163	0.360
Perceived Susceptibility	0.074	0.079	0.033	0.346
Perceived Benefit	-0.060	0.067	-0.267	-0.001

R Square = 0.872

## Discussion

This cross-sectional study aimed to analyze the self-protective behavior of VHVs in Southern Thailand during the COVID-19 outbreak. The study applied the Health Belief Model (HBM) to help interpret VHVs’ behavior in response to the pandemic. A total of 152 Thai VHVs participated in the study, predominantly female and Muslim, with a mean age of 49.2 years and low incomes. The majority of participants held leadership roles in COVID-19 screening within their villages, indicative of support from health providers that would be expected to positively influence their community engagement.

The demographic characteristics of the VHVs reflect the ethnic mix of Songkhla Province which is primarily Buddhist with a large Muslim minority. Ethnic composition of VHVs might be a factor in the success of VHVs at community engagement since cultural factors that could influence people’s compliance with health practices (e.g., the need to travel to religious events) differ between religious cultures. However, we did not formally test any hypotheses relating to this. The tendency for VHVs to be predominantly female has been observed in other studies, reflecting cultural norms and other factors, although the VHVs in our study were younger than those seen in other regions of Thailand (e.g. [11]). The only demographic characteristic that showed a clear statistically significant relationship with VHVs’ self-protective behavior in our study was their role, with Village Leaders having higher perceived compliance with self-protective behavior than non-leaders. This might reflect effects of VHV’s leaders’ greater experience but does suggest that an exploration of ways to empower non-leaders to improve their self-protective behavior could be helpful.

Our questionnaire showed that VHVs exhibited a positive perception of the HBM components related to social distancing and self-protective behavior during the COVID-19 pandemic. Consistent with other studies [32, 33], we found that VHVs acknowledged their susceptibility to contracting COVID-19 and recognized the benefit of self-protection, actively engaging in preventive measures by adhering to social distancing guidelines, following standard precautions, and complying with Thai Government policies. However, perceived barriers showed a weaker correlation with self-protective behavior, which has also been seen in similar studies [32, 34]. This suggests that the perceived obstacles, such as the inconvenience of wearing masks or practicing social distancing, did not impede VHVs’ engagement in self-protective behaviors. This might be explained by the extensive exposure of health volunteers to information about the severity of COVID-19 through various media sources and through their fieldwork experience of COVID-19 consequences that empowered them to tolerate perceived barriers in order to carry out their role. But the questionnaire answers relating to hand hygiene suggest that VHVs’compliance with hand hygiene measures may have been suboptimal, since VHVs reported lower perceived compliance with hand hygiene than with other barriers such as wearing masks. VHVs perceiving that they follow good self-protective behavior and have high self-efficacy despite suboptimal hand hygiene warrants further investigation, in case there is a need for greater emphasis on hand hygiene in VHVs’ training (e.g. in how to maintain frequent hand-washing of an adequate standard over an extended period of time, where there is a risk of fatigue with compliance).

We found that self-efficacy accounted for the majority of variance in VHVs’ COVID-19 self-protective behaviors, with the other HBM constructs providing limited explanation of the behaviors, which is consistent with previous research [18, 23, 35]. VHVs’ extensive experience, training, equipped status, community roles (including as role models), and strong health beliefs most likely explained their heightened sense of self-efficacy [36] which would also have been strongly influenced by VHVs’ required compliance with the Thai government’s Stop Covid policies.

As well as disseminating health information, VHVs also played a vital role in disease control through various surveillance activities, including home visits, fever screening, and monitoring at-risk groups’ movements. Their compliance with the Communicable Diseases Control Act and their volunteer role further contributed to their ability to engage in rigorous self-protection and disease prevention practices [37]. Social distancing was positively associated with VHVs’ self-protective behaviors, which aligns with previous studies emphasizing the importance of social distancing in preventive behaviors [38].

The study was conducted as a ‘live’ research investigation in response to the developing COVID-19 pandemic and has limitations. We focused on one geographic region, with a relatively small sample size that limited the power of our statistical tests to detect relationships between demographic variables and VHVs’ behaviors. The questionnaire relied on self-reported behaviors, with potential for recall bias. The cross-sectional study design precludes causal inferences, and we do not expect our findings to be generalizable to other populations or settings due to geographic variation of the cultural characteristics of the VHVs and the local communities that they engage with. It is also important to stress that the English translation of the questionnaire that we have provided for information (Appendix B) cannot exactly reflect how the Thai language questionnaire was implemented in practice, as we expect questionnaire interpretation and communication to be sensitive to the specific linguistic and cultural setting. However, our study has a number of strengths. The questionnaire was pre-planned and pilot-tested for validity and reliability. The researchers who administered the questionnaire received training and followed a standardized data collection process to help ensure consistency of the approach, to minimize heterogeneity. Furthermore, our strict data collection process ensured that there was no attrition, with all 152 recruited VHVs completing all 54 questions. This study was one of several independent studies conducted in Thailand during the COVID-19 pandemic exploring the roles of VHVs, most of which have so far only been published in the Thai language. Our study contributes to the wider research picture, helping to provide valuable insights into the efficacy of Thai VHVs’ self-protective behavior during the COVID-19 pandemic, with some suggestions for potential actions that might improve VHVs’ responses to any future pandemic.

## Conclusion

VHVs in Chana district of Songkhla Province perceived strong self-efficacy in their self-protective behaviors against COVID-19. This would be expected given that VHVs have been widely credited as having had an important role in mitigating the COVID-19 pandemic in Thailand. Our study suggests that self-efficacy may help to overcome barriers that might otherwise hinder behaviors to mitigate against COVID-19. However, our findings do suggest that compliance with hand hygiene might be a specific weakness for some VHVs that could be improved. We also found that compliance with perceived self-protective barriers was lower among some VHVs who were non-leaders, raising the question of whether VHVs in such roles could be empowered to improve their compliance in any future pandemic. Our relatively small sample size may not have been sufficient to detect other effects of demographic variables, although it is difficult to plan and conduct large studies at short notice in a pandemic. Nevertheless, this study contributes to the body of research on how VHVs interacted in their local communities in Thailand during the 2020 COVID-19 pandemic. Whilst the Health Belief Model provided a useful interpretational framework, VHVs’ self-protective behavior was primarily explained by self-efficacy.

### Electronic supplementary material

Below is the link to the electronic supplementary material.

Supplementary Material 1

## Data Availability

Data that support the findings of this study are provided in the paper and Appendices.
